# High-Oxygen-Affinity Hemoglobins—Case Series and Review of the Literature

**DOI:** 10.3390/jcm13020458

**Published:** 2024-01-14

**Authors:** Veroniki Komninaka, Pagona Flevari, Evangelia-Eleni Ntelaki, Eleni Yfanti, Theodoros Androutsakos, Ioannis Ntanasis-Stathopoulos, Evangelos Terpos

**Affiliations:** 1 Centre of Excellence in Rare Haematological (Haemoglobinopathies) & Rare Metabolic (Gaucher Disease) Diseases, Laiko General Hospital, 11527 Athens, Greece; verkomnin@gmail.com (V.K.); fpagona@yahoo.gr (P.F.); delaki@yahoo.com (E.-E.N.); elenifad@gmail.com (E.Y.); 2 Department of Pathophysiology, Medical School, National and Kapodistrian University of Athens, 11527 Athens, Greece; tandroutsak@med.uoa.gr; 3 Department of Clinical Therapeutics, School of Medicine, National and Kapodistrian University of Athens, 11528 Athens, Greece

**Keywords:** high-oxygen-affinity hemoglobins, Hb Crete, Hb Hiroshima, Hb Köln

## Abstract

Modifications of the hemoglobin (Hb) structure in regions involving the regulation of oxygen transport may lead to an increased oxygen affinity for the hemoglobin molecule and impaired oxygen delivery to the tissues. Herein, we present six patients with high-oxygen-affinity Hb variants, either in heterozygous form or in compound heterozygosity (such as heterozygosity for Hb Hiroshima, Köln, Crete, and compound heterozygosity Hb Crete with β or δβ thalassemia), in order to demonstrate the need for prompt and accurate diagnosis and enrich the limited literature due to the rarity of such cases. Hb Crete, Hb Hiroshima, and Hb Köln have distinct pathophysiologies and may result in different clinical phenotypes. In conclusion, high-oxygen-affinity hemoglobins are rare and inherited within a dominant autosomal manner, have various clinical presentations, and should always be suspected in patients with erythrocytosis. Their management (as phlebotomy or low-dose aspirin) should be based on an individualized assessment of the risk of complications, the medical history, concomitant symptoms, and quality of life.

## 1. Introduction

Hemoglobinopathies are a group of monogenic inherited disorders characterized by altered or absent/reduced hemoglobin (Hb) α or β chain synthesis, leading to thalassemia syndromes and structural hemoglobin variants. Abnormal hemoglobins caused by genetic mutations affecting the globin chains structure are called Hb variants. The modifications of the Hb structure in regions involving the regulation of oxygen (O_2_) transport may lead to an increased or reduced oxygen affinity for the Hb molecule and a subsequent impaired oxygen delivery to the tissues [1]. Low oxygen affinity hemoglobins deliver more oxygen to the tissues, reducing the erythropoietin production, and are sometimes associated with a right-shifted oxygen dissociation curve with increased P_50_.

High-oxygen-affinity variants are characterized by lower release of oxygen, leading to tissue hypoxia that, in turn, enhances erythropoietin production at the renal level, stimulating erythropoiesis. These variants may cause erythrocytosis (red cell mass greater than 125% above the expected value for sex and body mass) [2] and/or polycythemia (Hb > 16.5 g/dL − hematocrit (Hct) > 49% for men and Hb > 16.0 g/dL − Hct > 48% for women) [3]. 

The first case study of a high-oxygen-affinity hemoglobin variant was published by Charache et al. at 1966 [4]. To date, nearly 224 Hb variants with high oxygen affinity are known (see http://globin.bx.psu.edu/hbvar, HbVar database, accessed on 1 September 2023) [5], and one-third of them result in erythrocytosis [6].

## 2. Cases

In this case-based review, we present six patients with high-oxygen-affinity Hb variants in order to demonstrate their hematological and clinical features; to emphasize the need for prompt and accurate diagnosis; and to enrich the limited literature due to the rarity of such cases, commenting in parallel on the relevant literature. The study was approved by the institutional review committee of Laikon General Hospital (Ε15/ΔΓΝ02/9 September 2019).

### 2.1. Case 1

An 81-year-old female patient was referred to us for further investigation. The patient had been under previous hydroxyurea treatment for several years due to an incorrect diagnosis of myeloproliferative syndrome (although she was negative for the mutations of JAK2V617F and bcr/abl genes and did not meet WHO criteria for polycythemia vera). The complete blood count (CBC) revealed Hct 49.6%, Hb 16.2 g/dL, red blood cells (RBC) 6.84 M/μL, mean corpuscular volume (MCV) 72.5 fl, mean hemoglobin concentration (MCH) 23.7 pg, white blood cell count (WBC) 7.55 × 10^9^/L, and platelets (PLT) 226 × 10^9^/L. High-performance liquid chromatography (HPLC) analysis of Hb indicated the following fractions: Hb A 81.6%, Hb A_2_ 3.8%, and Hb F 3.7%, with no other variants being detected. The subsequent DNA analysis identified heterozygosity for the Hb Crete mutation CD129 G>C (Ala>Pro) (HGVS name: HBB:c.388G>C), a known high-oxygen-affinity Hb variant. The patient had no splenomegaly, no history of thrombosis, and reported no clinical symptoms.

### 2.2. Case 2

**Figure 1:**
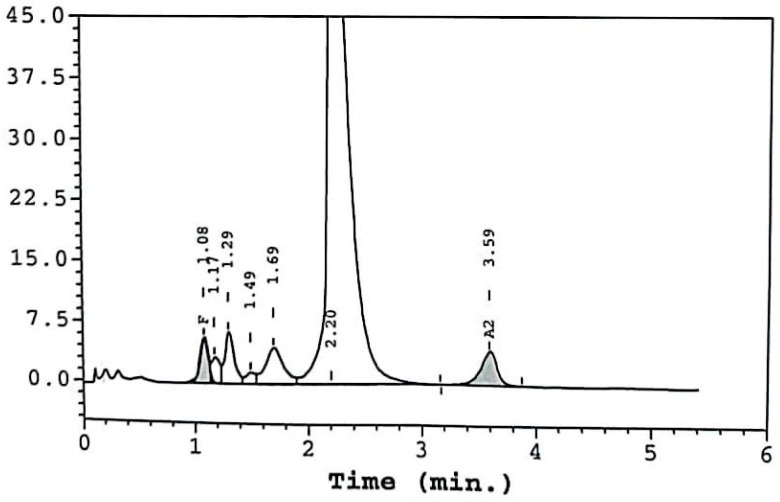
Case 2 HPLC results. Fractions in the vertical axis are expressed in %.

The daughter of case 1, a 50-year-old female, was also investigated due to the relevant family history and was diagnosed with compound heterozygoty for β thalassemia (β thal) and Hb Crete. The patient’s father was diagnosed with heterozygoty of β thal. The CBC revealed Hct 45.4%, Hb 15 g/dL, RBC 7.23 M/μL, MCV 62.8 fl, MCH 20.7 pg, WBC 9.49 × 10^9^/L, and PLT 308 × 10^9^/L. HPLC analysis of Hb indicated the following fractions: Hb A 59.3%, Hb A_2_ 4.3%, and Hb F 5.1%, with no other variants present (Figure 1). The subsequent DNA analysis identified the compound heterozygosity for Hb Crete and β thal (CD129 (G>C)/IVS-I-6 (T->C)). The patient had no clinical symptoms, no splenomegaly, and no history of thrombosis. Moreover, she reported a pregnancy without complications and the birth of a healthy child through caesarian section.

### 2.3. Case 3

A 15-year-old male was referred to us due to elevated RBC values. The CBC revealed Hct 41.6%, Hb 13.8 g/dL, RBC 6.48 M/μL, MCV 64.2fl, MCH 21.3 pg, WBC 5.59 × 10^9^/L, and PLT 195 × 10^9^/L. HPLC analysis of Hb indicated the following fractions: Hb A 65.1%, Hb A_2_ 2.1%, and Hb F 27.1%, with no other variant present. The DNA analysis identified compound heterozygosity for the Hb Crete and δβ thal mutations (CD129 (G>C)/δβ Sic). The patient had normal growth, reported no restrictions at activities, and had no splenomegaly or history of thrombosis.

### 2.4. Case 4

The sister of case 3, a 17-year-old female, was subsequently investigated due to the relevant family history. The CBC revealed Hct 39.4%, Hb 13.1 g/dL, RBC 5.64 M/μL, MCV 69.9 fl, MCH 23.2 pg, WBC 6.42 × 10^9^/L, and PLT 210 × 10^9^/L. HPLC analysis of Hb indicated the following fractions: Hb A 63.4%, Hb A_2_ 2.5%, and Hb F 29.2%, with no other variants present. DNA analysis identified compound heterozygosity for the Hb Crete and δβ thal mutations (CD129 (G>C)/δβ Sic). The patient had no splenomegaly, no history of thrombosis, and had normal growth and normal daily activities.

**Figure 2:**
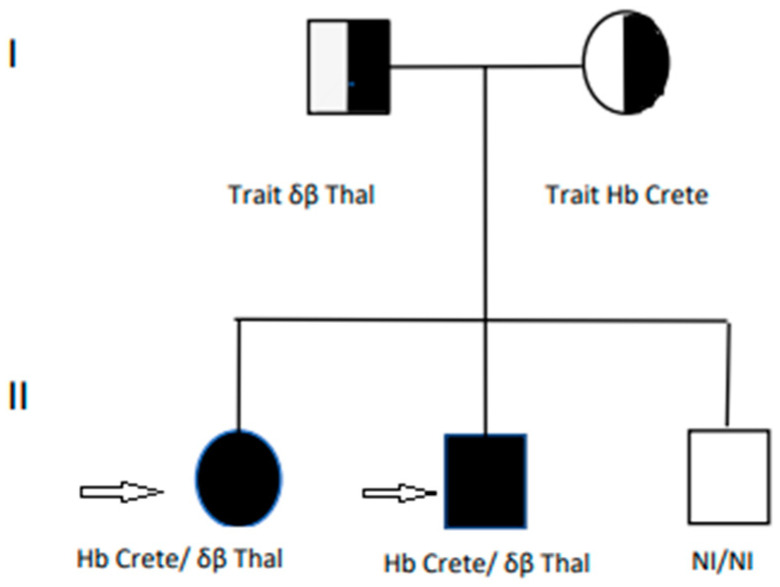
Family pedigree of cases 3 and 4 (arrows). (**I**) first generation, (**II**) second generation.

Cases 3 and 4 had another healthy sibling, while their parents were diagnosed with the traits of δβ thal and Hb Crete. The family pedigree of cases 3 and 4 is demonstrated in Figure 2. Cases 3 and 4 are marked by the arrows. Circles represent females and squares represent males. Semi-filled shapes indicate family members who were heterozygous for Hb Crete and δβ thal, while fully filled shapes indicate affected individuals. Unfilled shapes indicate normal (NI) individuals.

### 2.5. Case 5 

**Figure 3:**
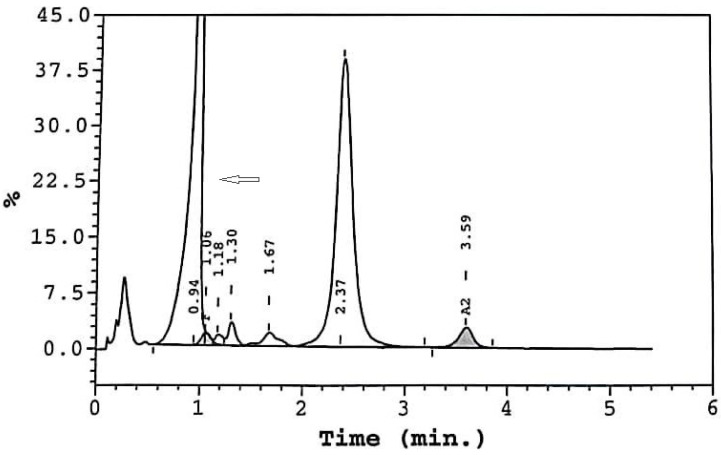
Case 5 HPLC results.

A 24-year-old female patient was referred to us due to eryhtrocytosis. The CBC revealed Hct 46.3%, Hb16 g/dL, RBC 5.18 M/μL, MCV 89.4 fl, MCH 30.9 pg, WBC 9.03 × 10^9^/L, and PLT 269 × 10^9^/L. HPLC analysis of Hb indicated the following fractions: Hb A 44.3%; Hb A_2_ 2.6%; Hb F 1.3%; and an unknown variant, Hb X 47.3% (Figure 3, arrow). The DNA analysis identified heterozygosity for Hb Hiroshima CD146 His>Asp (HBB:c.439 C>G), a known high-oxygen-affinity Hb variant. The patient experienced occasional mild headaches, but had no splenomegaly and no history of thrombosis.

### 2.6. Case 6

**Figure 4:**
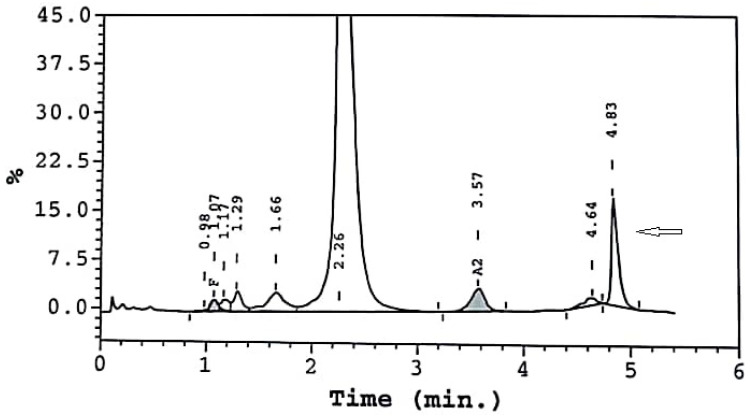
Case 6 HPLC results.

A 70-year-old male patient, who had already been diagnosed with heterozygoty for Hb Köln due to erythrocytosis, was referred to us for monitoring. The CBC revealed Hct 51.7%, Hb 15.2 g/dL, RBC 5.64 M/μL, MCV 91.7 fl, MCH 27 pg, WBC 9.37 × 10^9^/L, and PLT 140 × 10^9^/L. HPLC analysis of Hb indicated the following fractions: Hb A 80.2%; Hb A_2_ 3.3%; Hb F 0.9%; and an unknown variant, Hb X 7.3% (Figure 4, arrow). The DNA analysis identified heterozygosity for Hb Köln CD98 (G>A) (Val>Met) (HGVS name: HBB:c.295G>A), a known high-oxygen-affinity Hb variant. The patient reported occasional headaches, had splenomegaly, and had no history of thrombosis.

Table 1 summarizes the laboratory and clinical data of the aforementioned cases.

## 3. Discussion

Adult hemoglobin (Hb A) is a tetramer that binds oxygen tightly in the high-oxygen environment of the lungs and releases it to the tissues. This is demonstrated by the sigmoidal curve of the oxygen–hemoglobin dissociation curve. The p50 value is the partial pressure of oxygen at which the Hb molecule is half-saturated with oxygen. It is considered as a marker of changes at the oxygen–hemoglobin dissociation curve and should be used as a diagnostic tool for high-oxygen-affinity hemoglobins when available. A low p50 value is consistent with an Hb variant with increased oxygen affinity [6].

Most hemoglobinopathies show recessive inheritance, and high-oxygen-affinity Hb variants are inherited exceptionally by an autosomal-dominant pattern. Most reported patients are heterozygous (mutations in a single globin gene, Cases 1, 5, and 6). Homozygous patients have rarely been described and represent a more severe form of disease. 

The clinical presentation of patients with high-oxygen-affinity hemoglobins varies. Most of them are usually asymptomatic, with isolated and well-tolerated erythrocytosis on the hematology tests, or present a mild phenotype (Table 2) [7]. At times, the clinical course may be complicated by symptoms of hyperviscosity, such as mucosal erythema; headaches; vertigo; tinnitus; paresthesia in the extremities; and even, rarely, thrombotic events [6,8,9,10]. Our reported cases 1, 2, 3, and 4 had no symptoms, while cases 5 and 6 experienced mild, occasional headaches. Concomitant thalassemia may worsen the phenotype, as it decreases the amount of Hb A (cases 2, 3 and 4) and in turn increases the proportion of the high-oxygen-affinity Hb. In addition, compound heterozygotes for a high-affinity Hb variant and a thalassemic gene can present with severe erythrocytosis. The proportion of Hb F of case 2 (5.1%) was slightly higher for a β^+^ thalassemia trait, as were the Hb F proportions in cases 3 and 4 (27.1% and 29.2% respectively), who bore a Hb Crete gene and a δβ^0^-thalassemia gene. In both cases, the high Hb F values could have derived from the simultaneous presence of the Hb variant and thalassemia. Erythrocytosis is a result of the concurrent presence of a high Hb Crete percentage. It is possible that the type of thalassemia trait (β^0^, β^+^, δβ) could be the basic factor that modifies the phenotypes of patients with such compound heterozygosity. 

Nevertheless, we have to note that two-thirds of high-oxygen-affinity hemoglobins are not associated with erythrocytosis due to low-level expression of the Hb variant (e.g., presence of α globin chain mutation) or due to concomitant chronic hemolysis in the case of an unstable Hb variant. In approximately one-third of high-oxygen-affinity variants, the mutation may result in the production of an unstable hemoglobin molecule, and half of them are complicated by hemolysis, anemia, jaundice, and splenomegaly, covering erythrocytosis [11]. Due to the rarity of high-oxygen-affinity hemoglobins in compound heterozygosity with thalassemia, the underlying mechanisms that determine the phenotypes of the patients have yet to be fully understood.

It is important to make an early and correct diagnosis in order to avoid costly and unnecessary invasive diagnostic procedures, as well as inappropriate therapeutic interventions, and secondly, to provide diagnostic information and genetic counseling to the involved families. Case 1 was misdiagnosed with a myeloproliferative syndrome when referred to us, although the diagnosis of polycythemia vera did not meet the WHO criteria. As a result the patient underwent hydroxyurea treatment for several years due to erythrocytosis, a treatment which was not needed after the establishment of the Hb Crete heterozygoty diagnosis. 

The diagnostic workup includes: history, exclusion of other causes of erythrocytosis (cardiopulmonary disease, carbon monoxide poisoning, a myeloproliferative neoplasm, erythropoietin-producing tumor), pulse oxymetry measurement, hematology and biochemistry blood tests, normal or high serum erythropoietin (EPO) levels, low p50 value, HPLC electrophoresis, and genetic testing. 

There are high-oxygen-affinity Hb variants that are detected with a clear separation from Hb A in HPLC, while there are also some electrophoretically silent variants due to their neutral charge (such as Hb Crete, Figure 1). Capillary electrophoresis using the glycated hemoglobin (HbA1_C_) program may improve the separation of some Hb variants from Hb A, so it is better if both methods are performed in parallel. The final diagnosis is documented by gene testing. The diagnosis should also be suspected in patients with elevated HbA1c values if they have normal serum glucose measurements. In such cases, the only laboratory-significant anomaly is that of an unusually high HbA1c value [12].

Patients with high-oxygen-affinity hemoglobins accompanied by erythrocytosis usually have benign clinical courses without complications. Current management recommendations lack supporting evidence regarding the optimal management, so it should be individualized [7,11]. 

Smoking discontinuation [13] and physical activity should be encouraged. Regarding asymptomatic individuals, the preemptive phlebotomy practice lacks supportive evidence-based recommendations [6]. The use of phlebotomy should be individualized and only utilized in the case of the presence of hyperviscosity symptoms, since the resulting hematocrit and blood viscosity reduction may relieve headache, dizziness, and paresthesia and improve quality of life [7,14,15]. 

Administration of low-dose aspirin is also individualized depending on the thrombotic risk assessment [16]. It could be used for the prevention of thromboembolic complications in some patients with high-affinity variant Hbs under the same terms that it is administered to patients with polycythemia vera [6]. Patients with acute thromboembolic complications should be treated with antithrombotic agents according to the relevant guidelines.

Regarding our patients, none of them have required any therapeutic intervention up to now.

Increased morbidity or mortality have not been observed during pregnancy [17,18]. Case 2 corresponds to the data in the literature, as she gave birth to a living, healthy child and had an uncomplicated pregnancy. High-affinity hemoglobin variants identified on newborn screening are often asymptomatic in the newborn period [18].

## 4. Conclusions

High-oxygen-affinity hemoglobins are caused by rare β globin mutations; they are inherited in a dominant autosomal manner and may have variable clinical presentation. Understanding the pathophysiology of high-oxygen-affinity hemoglobins and being aware of their clinical and laboratory features may help clinicians in the differential diagnosis of erythrocytosis when no other underlying cause is identified, such as with myeloproliferative syndrome or other secondary causes. Their management should be based on an individualized assessment of the risk of complications, the medical history, concomitant symptoms, and quality of life. Our observations point out the need for continuous monitoring of such rare cases in order to produce evidence-based recommendations for patient management.

## Figures and Tables

**Table 1:** Laboratory and clinical data of the aforementioned cases.

	Reference Values (Female/Male)	1. Heterozygoty Hb Crete (Female, 81 Years Old)	2. Compound Heterozygoty Hb Crete/βthal (Female, 50 Years Old)	3. Compound Heterozygoty Hb Crete/δβthal, (Male, 15 Years Old)	4. Compound Heterozygoty Hb Crete/δβthal, (Female, 17 Years Old)	5. Heterozygoty Hb Hiroshima (Female, 24 Years Old)	6. Heterozygoty Köln (Male, 70 Years Old)
Hct (%)	36.0–45.0/ 42.0–52.5	49.6	45.4	41.6	39.4	46.3	51.7
Hb (gr/dL)	12–15.5/14–17	16.2	15.0	13.8	13.1	16.0	15.2
RBC (M/μL)	4.50–6.30	6.84	7.23	6.48	5.64	5.18	5.64
MCV (fl)	81.0–99.0	72.5	62.8	64.2	69.9	89.4	91.7
MCH (pg)	27.5–32.0	23.7	20.7	21.3	23.2	30.9	27
RDW-CV (%)	10.9–15.7	15.5	16.7	22.3	20.4	11.9	17.3
Hb A (%)		81.6	59.3	65.1	63.4	44.3	80.2
Hb A_2_ (%)	2.3–3.1	3.8	4.3	2.6	2.5	2.6	3.3
Hb F (%)	<2.0	3.7	5.1	27.1	29.2	1.3	0.9
Hb Χ variant		no	no	no	no	47.3	7.3
RBC morphology		abnormal	abnormal	abnormal	abnormal	normal	abnormal
LDH (U/L)	135–214	207	218	376	225	189	545
Total/ind bilirubin (mg/dl)	0.3–1.2/<0.7	(-)	0.94/0.65	2.03/1.46	1.12/0,71	0.41/0.17	3.87/2.62
Molecular findings		CD129 G>C/Normal	CD129 G>C/IVSI-n6	CD129 G>C/δβSic	CD129 G>C/δβSic	CD146His>Asp/Normal	CD295G>A/Normal
Phenotype		Hb Crete/Normal	Hb Crete/β^+^thal	Hb Crete/δβ^0^thal	Hb Crete/δβ^0^thal	Hb Hiroshima/Normal	Hb Koln/Normal
Splenomegaly		no	no	no	no	no	yes
Thrombosis history		no	no	no	no	no	no

Thal: thalassemia, Hct: hematocrit, Hb: hemoglobin, RBC: red blood cells, MCV: mean corpuscular volume, MCH: mean concentration of hemoglobin, RDW-CV: red cell distribution width, LDH: lactate dehydrogenase, ind: indirect.

**Table 2:** Laboratory and clinical features of Hb Crete, Hb Hiroshima, and Hb Köln.

	Mutation	RBC Morphology	Erythocytosis	Splenomegaly	P50	HPLC	Heat Instability Test	Arterial Blood Gas	Bohr Effect
Heterozygoty Hb Crete	CD129 G>C	abnormal	Mild	no	low	HbA, HbA2, HbF	positive	normal	normal
Heterozygoty Hb Hiroshima	CD146His>Asp	normal	Mild	no	low	Hb Hiroshima, HbA, HbA2, HbF	normal	Not available	reduced
Heterozygoty Köln	CD295G>A	abnormal	occasional	yes	low	Hgb Köln, HbA, HbA2, HbF	positive	normal	normal

## Data Availability

The data presented in the manuscript are available from the corresponding author upon reasonable request.
